# Optimal dynamic control approach in a multi-objective therapeutic scenario: Application to drug delivery in the treatment of prostate cancer

**DOI:** 10.1371/journal.pcbi.1006087

**Published:** 2018-04-19

**Authors:** Itziar Irurzun-Arana, Alvaro Janda, Sergio Ardanza-Trevijano, Iñaki F. Trocóniz

**Affiliations:** 1 Pharmacometrics & Systems Pharmacology group, Department of Pharmacy and Pharmaceutical Technology, School of Pharmacy and Nutrition, University of Navarra, Pamplona, Navarra, Spain; 2 IdiSNA, Navarra Institute for Health Research, Pamplona, Navarra, Spain; 3 Department of Physics and Applied Mathematics, University of Navarra, Pamplona, Navarra, Spain; Mount Sinai School of Medicine, UNITED STATES

## Abstract

Numerous problems encountered in computational biology can be formulated as optimization problems. In this context, optimization of drug release characteristics or dosing schedules for anticancer agents has become a prominent area not only for the development of new drugs, but also for established drugs. However, in complex systems, optimization of drug exposure is not a trivial task and cannot be efficiently addressed through trial-error simulation exercises. Finding a solution to those problems is a challenging task which requires more advanced strategies like optimal control theory. In this work, we perform an optimal control analysis on a previously developed computational model for the testosterone effects of triptorelin in prostate cancer patients with the goal of finding optimal drug-release characteristics. We demonstrate how numerical control optimization of non-linear models can be used to find better therapeutic approaches in order to improve the final outcome of the patients.

## Author summary

Mathematical models of the disease processes are widely used in computational biology to quantitatively describe the time course of disease progression and are often linked to pharmacokinetic–pharmacodynamic models in order to evaluate the effect of drug treatment on disease. Once the models are built from observed information and/or literature data, they can predict the dynamics of the system under different conditions through computer simulations. However, simulation exercises are not always effective to obtain the desired objectives due to the complexity of these systems. In this work, we optimized the release characteristics of a synthetic gonadotropin-releasing hormone analog used to induce chemical castration by inhibiting the testosterone levels in prostate cancer patients. The therapeutic goals to achieve were to minimize the initial flare up of testosterone levels and the time to reach testosterone values below castration limit, while maximizing the castration period of the patients. Our methodology, based on control theory, introduces a manipulable variable into the system’s equations to drive the model towards the established goals. We demonstrated how drug-release properties can be improved with the implementation of optimal control strategies to enhance the outcome of cancer patients. These methods are extrapolable to other problems encountered in the field.

## Introduction

Optimizing delivery systems targeting constant levels of drug concentration represents always a challenge for chronic diseases requiring continuous treatment and especially in those cases where the relationship between drug exposure (represented generally as levels of drug concentration plasma measured longitudinally) and pharmacological response is complex and non-linear. The management of prostate cancer with sustained release formulations of triptorelin (TRP) injected every 3-6 months represents a good example [1].

For the case of the hormone-sensitive prostate tumors the therapeutic goal of any pharmacology treatment is to maintain as longer as possible the levels of testosterone (TST) below the castration limit (CT) which is set to the plasma concentration value of 0.5 ng/mL [2].

**Fig 1:**
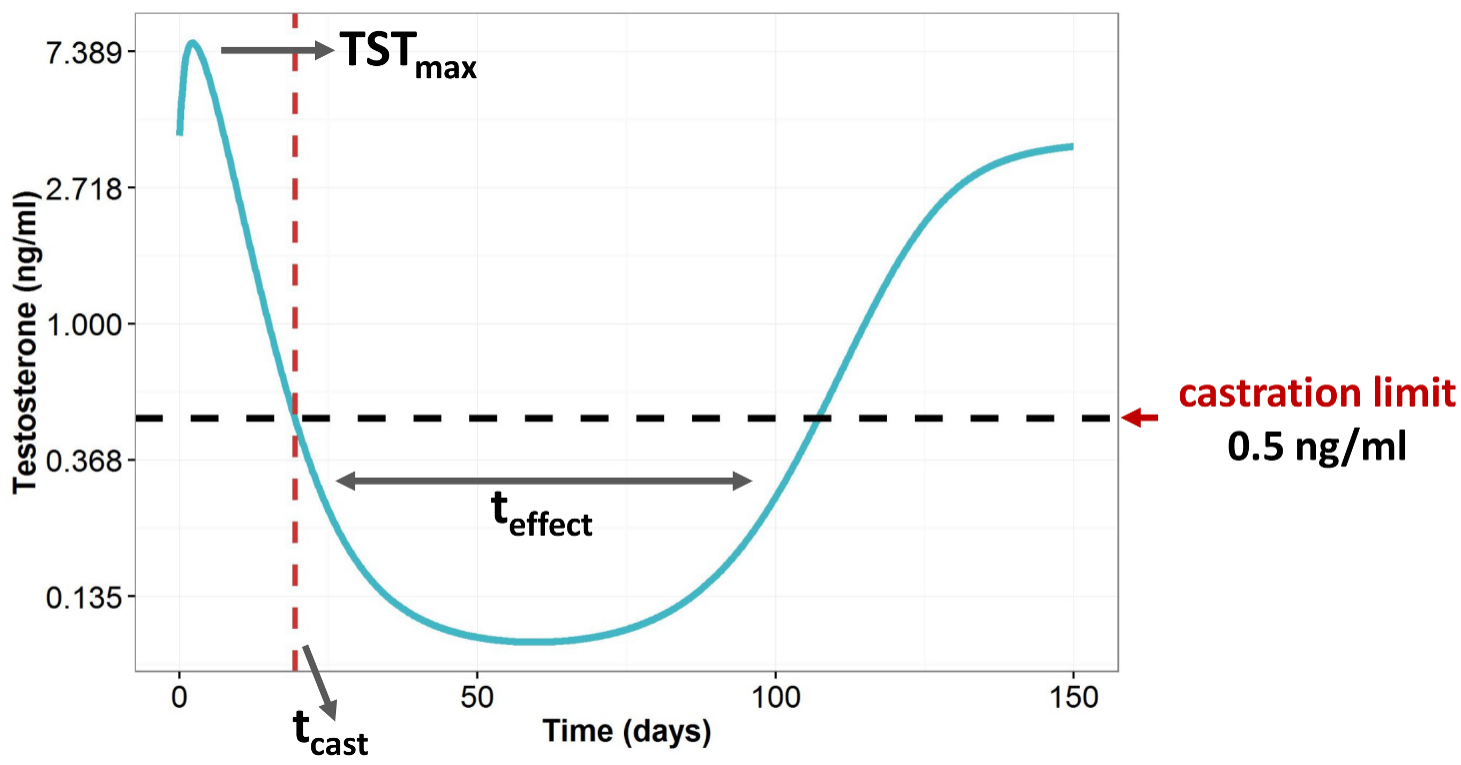
Typical testosterone profile after administration of triptorelin. TST_max_ refers to the maximal testosterone concentrations, t_cast_ indicates the time where testosterone levels fall below 0.5ng/ml (castration limit of prostate cancer patients, marked with an horizontal dashed line in the figure) and t_effect_ indicates the castration period of the patients.

**Fig 2:**
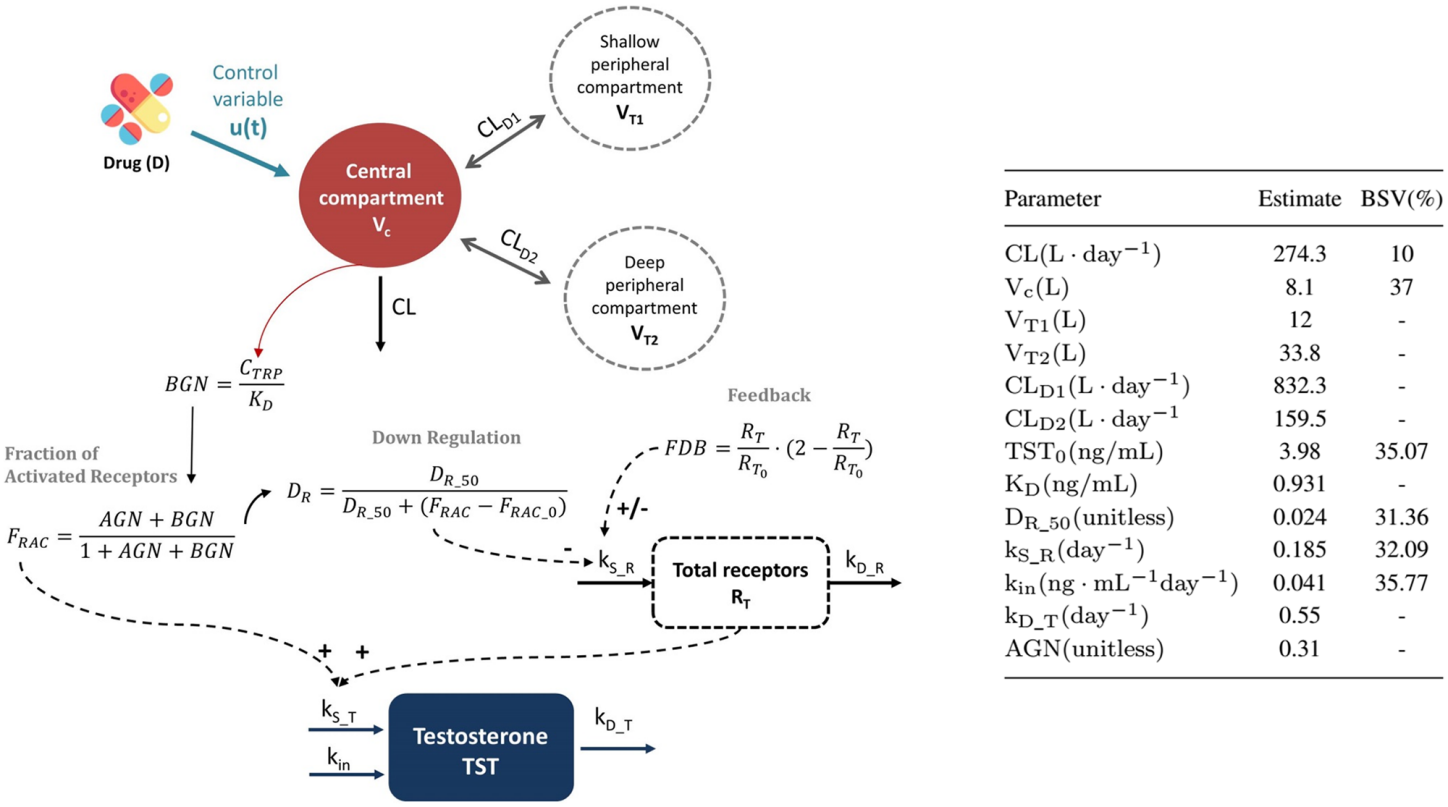
Schematic representation of the state variables and control input for the pharmakinetic-pharmacodynamic model of the testosterone effects of Triptorelin (left) and model parameter estimates (right). C_TRP_, serum concentrations of Triptorelin; CL, apparent total clearance; V_c_, V_T1_, and V_T2_, apparent volumes of distribution of the central, shallow, and deep peripheral compartments, respectively; CL_D1_ and CL_D2_, distribution clearances between the central and peripheral compartments; R_T_, total receptors; R_T_0__, total receptors at baseline; TST_0_, baseline testosterone level; K_D_, receptor equilibrium dissociation constant of triptorelin; D_R_, down-regulation process; D_R_50_, the value that elicits a 50% maximal reduction in k_S_R_ for a given amount of total receptors; k_S_R_, zero-order rate constants of receptor synthesis; k_D_R_, first-order rate constants of degradation; k_S_R_, zero-order rate constants of testosterone synthesis; k_D_T_, first-order rate constants of testosterone degradation; k_in_, zero-order rate production of testosterone independent from gonadotropins; AGN, ratio between the endogenous agonist concentration and its receptor equilibrium dissociation constant; FDB, feedback.

In recent past, we have developed a mechanistic computational model for the TST effects of the agonist TRP in prostate cancer patients using longitudinal pharmacokinetic (PK; drug concentration in plasma) and pharmacodynamics (PD; TST concentrations in plasma) data obtained from several clinical trials testing the efficacy of different sustained-release formulations (SR) [3]. Briefly, TRP exerts its action by increasing the fraction of activated receptors and therefore stimulating the production of TST. However, the prolonged exposure of TRP causes receptor down-regulation, resulting in a reduced synthesis of TST. The typical TST vs time profile after a single injection of TRP is represented in Fig 1. The schematic representation of the PKPD model developed for TST effects of TRP, excluding the absorption compartments of the original model, and the estimates of model parameters are shown in Fig 2.

As highlighted in Fig 1 there are three critical aspects to be taken into consideration at the time to develop an innovative delivery system of TRP for the treatment of prostate cancer: initial flare up, time to reach CT, and castration period. Ideally, such new formulation should release TRP at a rate eliciting levels of concentration in plasma minimizing both the initial flare up and the time to reach CT, as well as maximizing the castration period. Specifically, limitation in the TST flare-up (TST_max_) to 50% increase with respect to baseline, minimize time to castration after first injection (t_cast_) to values below 3 weeks, and extend the castration time after injection (t_effect_) for at least 9 months.

Given the complex relationship between concentrations of TRP in plasma and response as represented in Figs 1 and 2, together with the requisite of maintaining the TST profiles within the constraints mentioned above, optimization of the rate of drug release is not a trivial task and cannot be efficiently addressed through an extensive trial & error simulation exercise.

In the current work we aimed to optimize the release profile of TRP from SR formulations matching the multi-objective therapeutic needs applying optimal control methodology [4]. The rationale behind the decision of focusing on the release process is based on the assumption that once the drug is absorbed and reaches systemic circulation (represented as part of the central compartment in Fig 2) it follows the same distribution and elimination characteristics regardless the type of formulation administered. The same is assumed with respect to the TST response, the rate of synthesis and degradation of TST and receptors, the dynamics of receptor occupation, and the down-regulation process. These mechanisms are independent from the absorption properties of the drug.

Despite we focused on a specific case, the workflow and methodology used can be readily translate to other therapeutic areas and scenarios such as dosing schedule optimization and personalized treatments.

## Materials and methods

**Fig 3:**
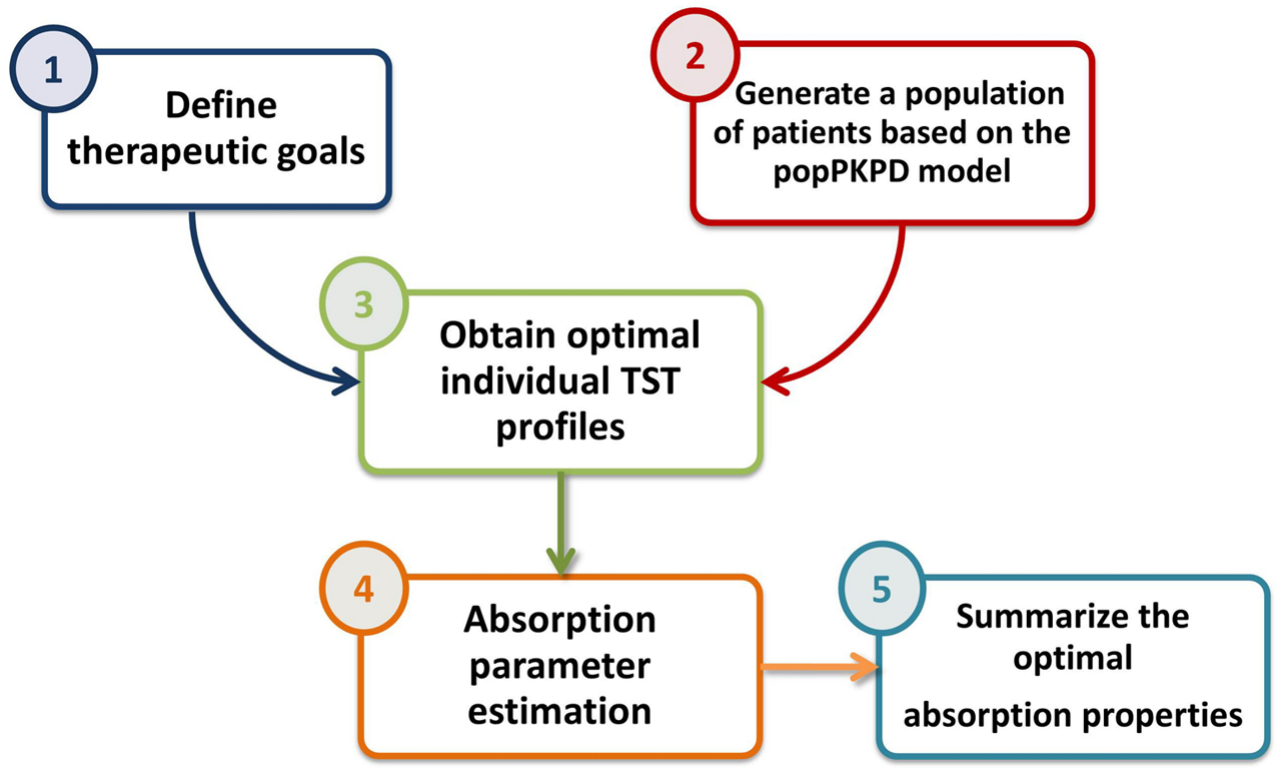
Principal steps implemented in our methodology.

Having defined the therapeutic goals of the project, the analysis was divided in several steps: i) a population of virtual subjects were generated in order to have a representative population of the study; ii) the optimal TST profiles for each virtual patient were derived by means of optimal control methods; and finally iii) the empirical absorption profiles obtained in step ii were characterized using parametric models to assist biopharmaceutics at the time to develop and evaluate new SR formulations of triptorelin. A schematic representation of the workflow is given in Fig 3.

### Generation of a virtual patient population

Values listed in the table inserted in Fig 2 include estimates of typical population parameters and between-subject variability (represented by BSV in the table and hereafter) obtained from [3] for a set of model parameters. In order to obtain the population of virtual patients, parameters were modelled as $P_{i}=P_{pop}\times e^{\eta_{i\_P}}$, where *P*_*i*_ and *P*_*pop*_ represent the *i*^*th*^ individual and typical population values of the *P* parameter, respectively, and *η*_*i*_*P*_ corresponds the deviation of *P*_*i*_ with respect the typical value *P*_*pop*_; the set of individual *η*_*i*_*P*_ forms a random variable with mean value of 0 and variance $\omega_{P}^{2}$ following a normal distribution, whereas the distribution of individual parameters is log-normal. The magnitude of $\omega_{P}^{2}$ reflects the BSV associated to a specific model parameter, which in Fig 2 is expressed as coefficient of variation (CV%).

One thousand set of disposition (clearance and volume of distribution in the central compartment, represented as CL and V_c_ respectively), pharmacodynamics (receptor equilibrium dissociation constant of triptorelin (K_D_)) and system (baseline TST levels (TST_0_), zero-order rate of TST production independent from gonadotropins (k_in_), zero-order rate constants of receptor synthesis (k_S_R_) and the value that elicits a 50% maximal reduction in k_S_R_ for a given amount of total receptors (D_R_50_)) related parameters were generated using the typical population estimates and corresponding marginal distributions reported in the table of Fig 2. The parameter values for the virtual population were generated with NONMEM 7.2 [5].

### Optimal control

An optimal control problem is a dynamic optimization problem in which the state of a system is linked in time to the application of a control function *u*(*t*), which drives the system towards a desirable outcome by minimizing a cost function *J*(*u*) subject to operating constraints [4, 6]. In other words, the control variable *u*(*t*) forces a system to have an optimal performance. The concrete control strategy will depend upon the criterion used to decide what is meant by “optimal”; in the current case TST_max_ < 1.5 ⋅ TST_0_ ng/ml, minimize t_cast_ ≤ 3 weeks, and maximize t_effect_ ≥ 9 months.

Therefore any optimal control problem can be formulated to find the magnitude of *u*(*t*) over the time of study [from initial time *t*_0_ to final time *t*_*f*_] such that:

$$\begin{array}{c} \begin{array}{cccc} & \underset{u(t)}{\text{min}} & & J\left(u\right)=\phi \left[x\left(t_{f}\right)\right]+\int_{t_{0}}^{t_{f}}L\left[x\left(t\right),u\left(t\right)\right]dt\\ & \text{subject}\;\text{to} & & \frac{dx(t)}{dt}=f\left(x\left(t\right),u\left(t\right),t\right)\\ & & & x\left(t_{0}\right)=x_{0},\\ & & & h(x(t),u(t))=0,\\ & & & g(x(t),u(t))\leq 0, \end{array} \end{array}$$ (1)

where *J*(*u*) is the cost function, *u*(*t*) is the control variable; *x*(*t*) the vector of state variables; *x*_0_ the set of initial conditions of the state variables; *h*() the equality constraints; and *g*() the inequality constraints. The general form of the equation in *J*(*u*) is known as Bolza optimization problem [7], which is represented as the sum of a terminal cost functional (Mayer problem) and an integral function of the state and control from *t*_0_ to *t*_*f*_ (Lagrange problem). For a more detailed information see S1 Text.

Fig 2 shows a schematic representation of the state variables and control input defined in this work. The state system is characterized by the variables that predict serum concentrations of triptorelin (C_TRP_), concentrations of triptorelin in the shallow and deep peripheral compartments (C_1_ and C_2_ respectively), drug input profile (D), amount of total receptors (R_T_), and optimal testosterone levels (TST), each of them represented by the corresponding ordinary differential equation as shown in Table 1. Note that in Fig 2 the terms resembling the 0^*th*^ and 1^*st*^ order absorption processes have been removed from the original model structure from [3] and have been replaced by the new control variable *u*(*t*). Therefore the expression associated to the rate of change of the levels of TRP in plasma ($\dot{C_{\text{TRP}(t)}}$) is:

$$\begin{array}{c} \begin{array}{c} \dot{C_{TRP}}=u\left(t\right)+\frac{CL_{D1}}{V_{T1}}\cdot c_{1}\left(t\right)+\frac{CL_{D2}}{V_{T2}}\cdot c_{2}\left(t\right)-\frac{CL_{D1}}{V_{c}}\cdot C_{TRP}\left(t\right)\\ -\frac{CL_{D2}}{V_{c}}\cdot C_{TRP}\left(t\right)-\frac{CL}{V_{c}}\cdot C_{TRP}\left(t\right) \end{array} \end{array}$$ (2)

An additional compartment *D* was defined, where the dose of TRP administered to the patients (10mg in this evaluation exercise) was placed as initial condition (*D*_0_). The control variable *u*(*t*) leaves this compartment and enters to the central (systemic) compartment of TRP as follows:

$$\begin{array}{c} \dot{D}=-u\left(t\right) \end{array}$$ (3)

Recall that, *u*(*t*) (ng/day) does not represent any particular mechanism of absorption (i.e., zero and/or first order kinetics), but a vector of different values that influence the system to behave in a pre-determined (optimal) way.

**Table 1:** Summary of the setup of the different components of the optimal control problem.

	Phase I, *t* ∈ [0, t_cast_]	Phase II, *t* ∈ [t_cast_,280+t_cast_]
Cost function	*J*_*I*_(*u*) = *t*_*cast*_	$J_{II}\left(u\right)=\int_{t_{cast}}^{280+t_{cast}}TST{\left(t\right)}^{2}dt$
State variables	subject to:	
	$\dot{D}=-u\left(t\right)$	
	$\dot{C_{1}}=-\frac{{\text{CL}}_{D1}}{V_{T1}}\cdot c_{1}\left(t\right)+\frac{{\text{CL}}_{D1}}{V_{c}}\cdot C_{\text{TRP}}\left(t\right)$	
	$\dot{C_{2}}=-\frac{{\text{CL}}_{D2}}{V_{T2}}\cdot c_{2}\left(t\right)+\frac{{\text{CL}}_{D2}}{V_{c}}\cdot C_{\text{TRP}}\left(t\right)$	
	$\dot{C_{\text{TRP}}}=u\left(t\right)+\frac{{\text{CL}}_{D1}}{V_{T1}}\cdot c_{1}\left(t\right)+\frac{{\text{CL}}_{D2}}{V_{T2}}\cdot c_{2}\left(t\right)-\frac{{\text{CL}}_{D1}}{V_{c}}\cdot C_{\text{TRP}}\left(t\right)-\frac{{\text{CL}}_{D2}}{V_{c}}\cdot C_{\text{TRP}}\left(t\right)-\frac{\text{CL}}{V_{c}}\cdot C_{\text{TRP}}\left(t\right)$	
	$\dot{R_{T}}=k_{S\_R}\left(D_{R}\cdot \text{FDB}\right)-k_{D\_R}\cdot R_{T}\left(t\right)$	
	$\dot{\text{TST}}=k_{S\_T}\left(\frac{\text{AGN}+\text{BGN}}{1+\text{AGN}+\text{BGN}}\cdot R_{T}\left(t\right)\right)+k_{\text{in}}-k_{D\_T}\cdot \text{TST}\left(t\right)$	
Initial conditions		
	$D_{{}_{I}}\left(t_{0}\right)=\text{Dose}$	$D_{{}_{\text{II}}}\left(t_{0}\right)=D_{{}_{I}}\left(t_{\text{cast}}\right)$
	$C_{1_{I}}\left(t_{0}\right)=0$	$C_{1_{\text{II}}}\left(t_{0}\right)=C_{1_{I}}\left(t_{\text{cast}}\right)$
	$C_{2_{I}}\left(t_{0}\right)=0$	$C_{2_{\text{II}}}\left(t_{0}\right)=C_{2_{I}}\left(t_{\text{cast}}\right)$
	$C_{{\text{TRP}}_{I}}\left(t_{0}\right)=0$	$C_{{\text{TRP}}_{\text{II}}}\left(t_{0}\right)=C_{{\text{TRP}}_{I}}\left(t_{\text{cast}}\right)$
	$R_{T_{I}}\left(t_{0}\right)=1$	$R_{T_{\text{II}}}\left(t_{0}\right)=R_{T_{I}}\left(t_{\text{cast}}\right)$
	${\text{TST}}_{{}_{I}}\left(t_{0}\right)={\text{TST}}_{0}$	${\text{TST}}_{{}_{\text{II}}}\left(t_{0}\right)={\text{TST}}_{{}_{I}}\left(t_{\text{cast}}\right)$
Inequality constraints (Boundaries)		
	D ∈ [0, Dose]	
	${\text{TST}}_{{}_{I}}\in \left[0.5,1.5\cdot {\text{TST}}_{0}\right]$	${\text{TST}}_{{}_{\text{II}}}\in \left[0,0.5\right]$
Equality constraints		
	${\text{TST}}_{{}_{I}}\left(t=t_{\text{cast}}\right)-0.5=0$	

where $\text{BGN}=\frac{C_{\text{TRP}}}{K_{D}}$, $\text{FDB}=\frac{R_{T}}{R_{T\_0}}\cdot \left(2-\frac{R_{T}}{R_{T0}}\right)$, R_T_0__ = 1, $D_{R}=\frac{D_{R\_50}}{D_{R\_50}+\left(\frac{\text{AGN}+\text{BGN}}{1+\text{AGN}+\text{BGN}}-\frac{\text{AGN}}{1+\text{AGN}}\right)}$, Dose = 10mg and *t*_0_ makes reference to the initial time. The rest of the parameters have been already defined in Fig 2. The subscript _I_ and _II_ in the model parameters refers to the first and second phase respectively.

The aim of this work was to find the time profile of *u*(*t*) (input function of TRP into the central compartment) that minimizes an objective (or cost) function and satisfies all constraints which represent the boundaries and therapeutic goals to be achieved (see Table 1). The choice of an objective function represents a critical aspect in optimal control problems [8]. Here, the problem is divided into two phases each represented by a different cost function and defined between: (i) 0 and t_cast_, and (ii) t_cast_ and ≥ 280 + t_cast_ days, respectively.

During the first phase (from 0 to t_cast_) the *u*(*t*) profile is optimized to transfer the system from an initial state TST_0_(baseline testosterone level) to the final state of 0.5ng/ml (CT value) in the shortest possible time. To solve the first phase of the optimization problem, the following objective function and equality constraint were defined respectively:

$$\begin{array}{c} J_{I}\left(u\right)=t_{cast} \end{array}$$ (4)

$$\begin{array}{c} TST(t=t_{cast})-0.5=0 \end{array}$$ (5)

where t_cast_ is an static control variable for minimizing *J*_*I*_. Here, we wished to obtain the minimum value of t_cast_ that causes TST levels to achieve the CT value. The final time t_cast_ was not known in advance, and that is the reason why the optimization problem was divided into two different phases.

Additionally, an inequality constraint was added to limit the initial flare-up of the testosterone below 50% increase with respect to baseline:

$$\begin{array}{c} TST\left(t\right)<1.5\cdot TST_{0} \end{array}$$ (6)

The second phase, covering the period between t_cast_ and 280+t_cast_ days, aims to maintain the TST levels below CT. If a second objective function or constraints were not incorporated into the optimization problem, values of TST rose above CT at times much earlier than 280 days. The approach used to overcome the above mentioned undesired effect and maintain TST predictions within the therapeutic goal led to the minimization of a second objective function of the form:

$$\begin{array}{c} J_{II}\left(u\right)=\int_{t_{cast}}^{280+t_{cast}}TST{\left(t\right)}^{2}dt \end{array}$$ (7)

The rationale for formulating *J*_*II*_ using a quadratic function (*TST*(*t*)^2^) for minimizing testosterone levels, instead of *TST*(*t*), was because it offers relevant mathematical advantages in the context of optimization problems. In optimal control theory, one of the main necessary conditions for optimality is that control variables minimize a Hamiltonian function over *u*(*t*). The Hamiltonian becomes convex if quadratic forms are used for the objectives and thus the problem will have a unique minimizer [4]. See S1 Text for more information about the Hamiltonian matrix and the necessary and sufficient conditions for optimal control problems. Furthermore, using squared terms amplify the effects of large variations and de-emphasize the contributions of small fluctuations.

Continuity between the two phases of the optimization problem was ensured by imposing the initial conditions of the state variables at phase II to be equal to their final values at the end of the phase I (see Table 1).

Alternatively, other objective functions or constraints could have been defined. For example, an alternative approach to model the second phase of the optimal control problem is to only add the inequality constraint TST[t_cast_: (280 + t_cast_)] − 0.5 < 0, instead of a second objective function *J*_*II*_. This approach resulted in TST levels closer to CT compared to the values obtained with the addition of *J*_*II*_. However, in the work from [1, 9] suggested that a CT value lower than 0.2 ng/ml could be an even better target to maximize therapeutic outcomes of prostate cancer patients. Due to these variations in the definition of the most appropriate CT value, we prioritized the minimization of TST levels during the second phase using *J*_*II*_ because we obtained the lowest possible values of TST.

Table 1 summarizes the setup of the different components of the optimal control problem described above. There exists different methods to solve this type of problems [10, 11]. In our case, the dynamic optimization problem was solved numerically via direct methods with the IPOPT Solver (Interior Point OPTimizer) [12] which is freely available in the APMonitor Optimization Suite (http://apmonitor.com/) through MATLAB programming environment [13]. The results were evaluated by calculating the proportion of individuals that achieved the described therapeutic goals and constraints.

For more information about control theory see the works from [6, 8, 14] and for a more comprehensive overview of the role of optimal control in cancer research read the reviews from [4, 10, 15].

### Mechanistic characterization of the optimal absorption profiles

During the optimal control exercise, values of TST in plasma were obtained approximately every 12h for the first phase and every 120h in the second phase. Given the fact that the disposition, pharmacodynamics, and system parameters were already known as they were randomly generated as described in Material and methods, the analyses of the TST profiles described in this section focused on the mechanistic/parametric characterization of the absorption process of TRP aiming to provide biopharmaceutics with metrics useful to guide the development of new sustained release formulations. Those metrics are the fractions of the total dose injected absorbed following 0^*th*^ and 1^*st*^ order processes, the cumulative drug release profiles over time, the percentage of the dose that should remain in the site of injection at t_cast_ and the time at which the different absorption mechanism are activated.

The absorption model used to estimate the corresponding absorption parameters allowing afterwards computation of metrics is represented in the work from [3] and comprises three non-simultaneous absorption mechanisms, two of them following 1^*st*^ order kinetics and the third one following a 0^*th*^ order process. This model is considered of a sufficient complexity to deal with almost any absorption profile that can take place after administration of SR formulations [16, 17]. A schematic representation of the structural model with the corresponding ordinary differential equations is provided in S1 Fig.

The analyses were performed with the NONMEM version 7.2 software [5], following a two stage approach in which the parameters of each subject are first obtained and summary statistics (median, and 95th confidence intervals) are then calculated. BSV in the absorption parameters was modelled exponentially as described in section for the rest of model parameters. TST concentrations obtained in step were logarithmically transformed for the analysis, and residual variability was modeled by using an additive error model on log-transformed data.

## Results

### Optimal pharmacodynamic profiles

**Fig 4:**
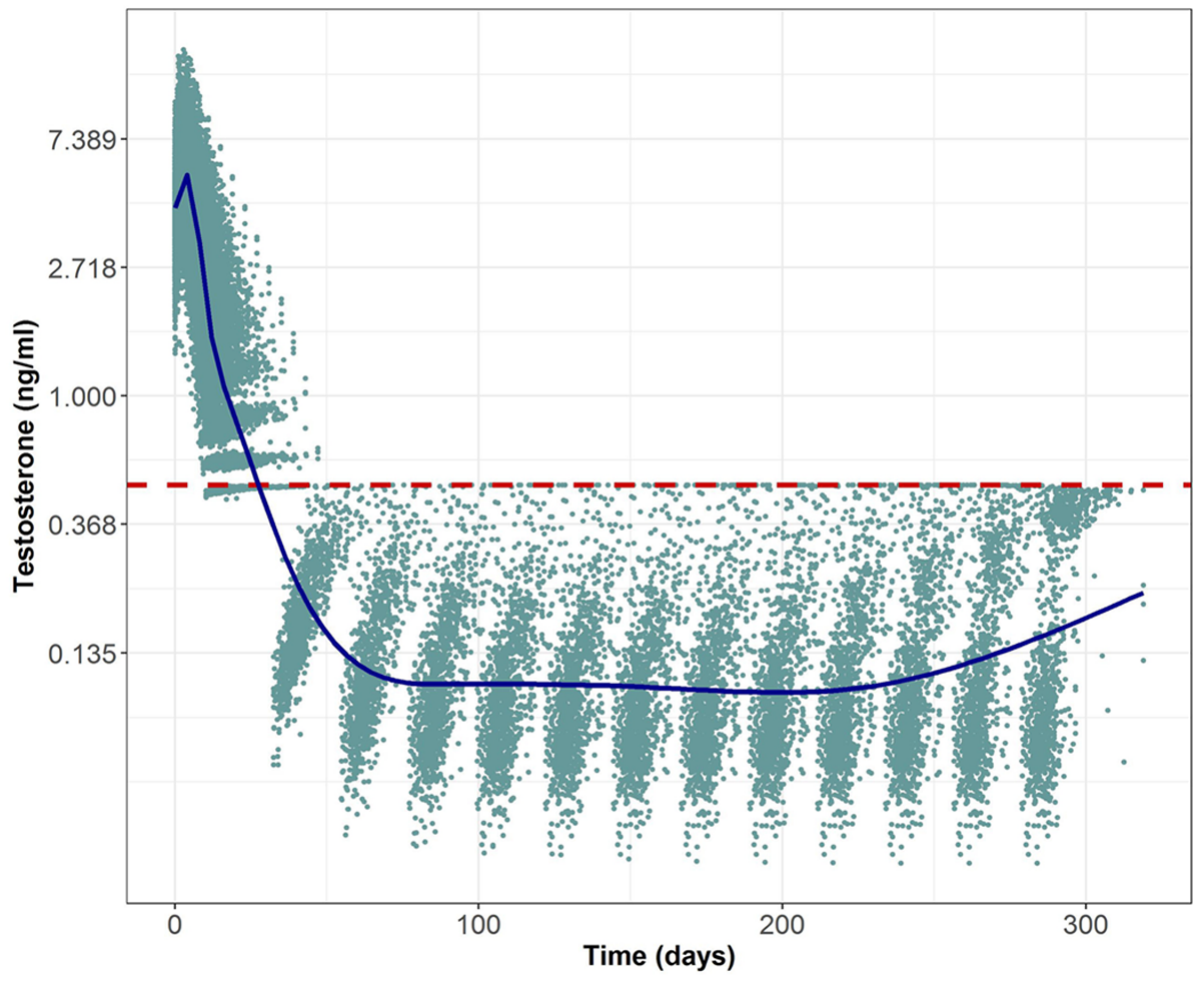
Optimal testosterone (TST) profiles for 1000 simulated individuals. Solid circles represent optimal TST observations obtained after the optimal control approach, solid line represent the median tendency of the data and red dashed line indicates the castration limit (0.5ng/ml) of prostate cancer patients.

Fig 4 (blue points) illustrates the optimal testosterone profiles for the 1000 hypothetical individuals that we obtained after applying the optimal control problem formulated in Table 1. The initial dose was considered to be 10mg. The code and data to reproduce these results in MATLAB can be found in the S1 Data. All of them achieved the 3 quantitative therapeutic goals (95% interval confidence between parenthesis) defined in the Introduction section: time to castration was minimized to 18.96 days (11.408—36.289), the increase of TST levels at the flare was always smaller than 50% with respect to baseline (36.8%-50.002%), and t_effect_ was greater than 280 for all the patients.

These profiles were generated with the manipulable variable *u*(*t*) which could take any values in order to minimize the multi-objective problem. However if we looked to the TRP concentration vs time profiles that induced the optimal TST levels (data not shown), those profiles did not seem attainable by using simple first or zero order kinetics. That was the reason to directly approximate the TST levels with the PKPD model presented by [3] and estimate the most adequate absorption parameters.

### Optimal release characteristics

**Fig 5:**
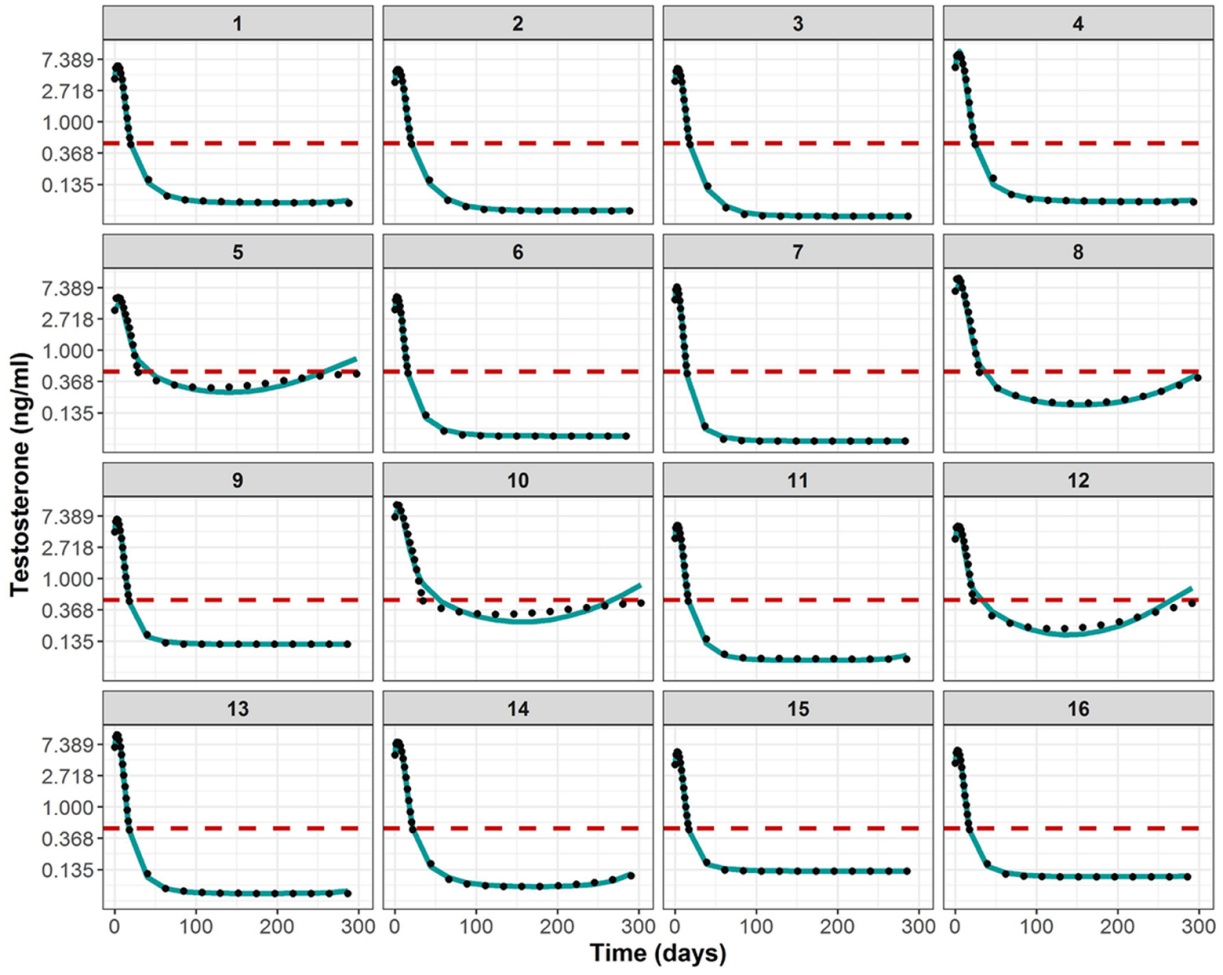
Optimal testosterone profiles of the 1000 virtual patients. Optimal testosterone observations (solid circles) with individual predictions (solid blue lines) of the pharmacokinetic/pharmacodynamic model and a red dashed line indicating the castration limit (0.5ng/ml) of prostate cancer patients.

**Fig 6:**
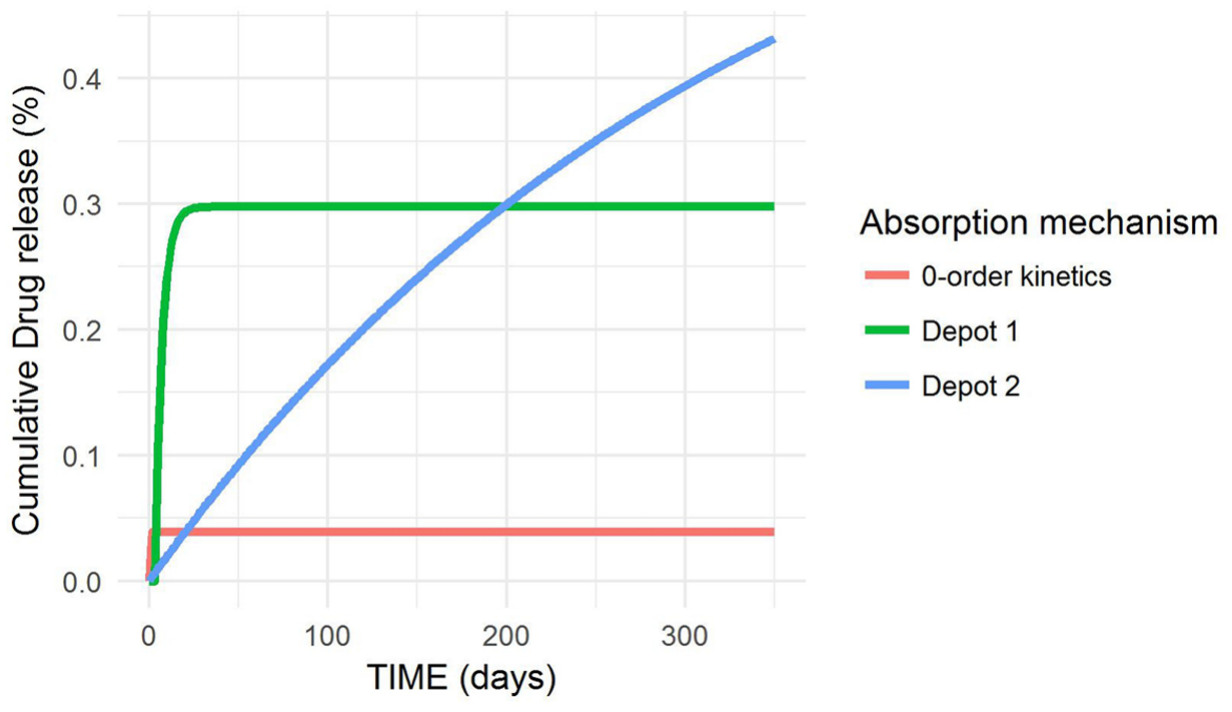
Optimal drug release characteristics following each of the absorption mechanisms.

The optimal release characteristics corresponding to the selected PKPD model from [3] are listed in Table 2. The final model adequately described the optimal TST profiles calculated in the previous section as shown in the individual profiles of Fig 5. A lag time was associated with one absorption compartment. The first order rate constant of absorption of the second depot compartment (K_A2_) had a very low median value (0.003 day^−1^), resulting in a slow decay of TRP in serum concentrations. The first order rate constant of the first depot compartment (K_A1_), instead, had a higher value (0.25 day^−1^) to allow for a rapid decay of the TST levels in the firsts days of treatment. Table 2 also indicates that most of the drug is released following 1^*st*^ order kinetics as the fraction of drug associated with the 0^*th*^ order absorption process (F_inf_) is very small (4%). This result is also reflected in Fig 6, where the median tendency of the drug release following each of the absorption mechanisms for the 1000 individuals is shown. The values of the duration of the 0^*th*^ order process (D_inf_) varied immensely between individuals (from hours to more than 200 days), thus the variability term was removed from this parameter.

**Table 2:** Population absorption parameters estimated for the optimal triptorelin profiles. The median values and the 95% confidence intervals (CI%) are shown for a population of 1000 patients.

Parameter	Estimate (CI%)
D_inf_ (day)	1.66 (-)
K_A1_(day^−1^)	0.25 (0.108-0.502)
K_A2_(day^−1^)	0.003 (0.0014-0.006)
F_1_	0.298 (0.067-0.6)
F_2_	0.664 (0.378-0.923)
F_inf_	0.039 (7.85e-05-0.07)
t_lag_ (day)	3.3 (2.336-5.188)

where D_inf_ is the duration of the zero-order absorption process, K_A1_ and K_A2_ are the first order rate constants of the first and second depot compartments respectively, F_1_, F_2_ and F_inf_ represent the fraction of the drug associated with the first and second depot compartments and the zero-order absorption process respectively, and t_lag_ is the lag time associated to the first absorption compartment.

**Fig 7:**
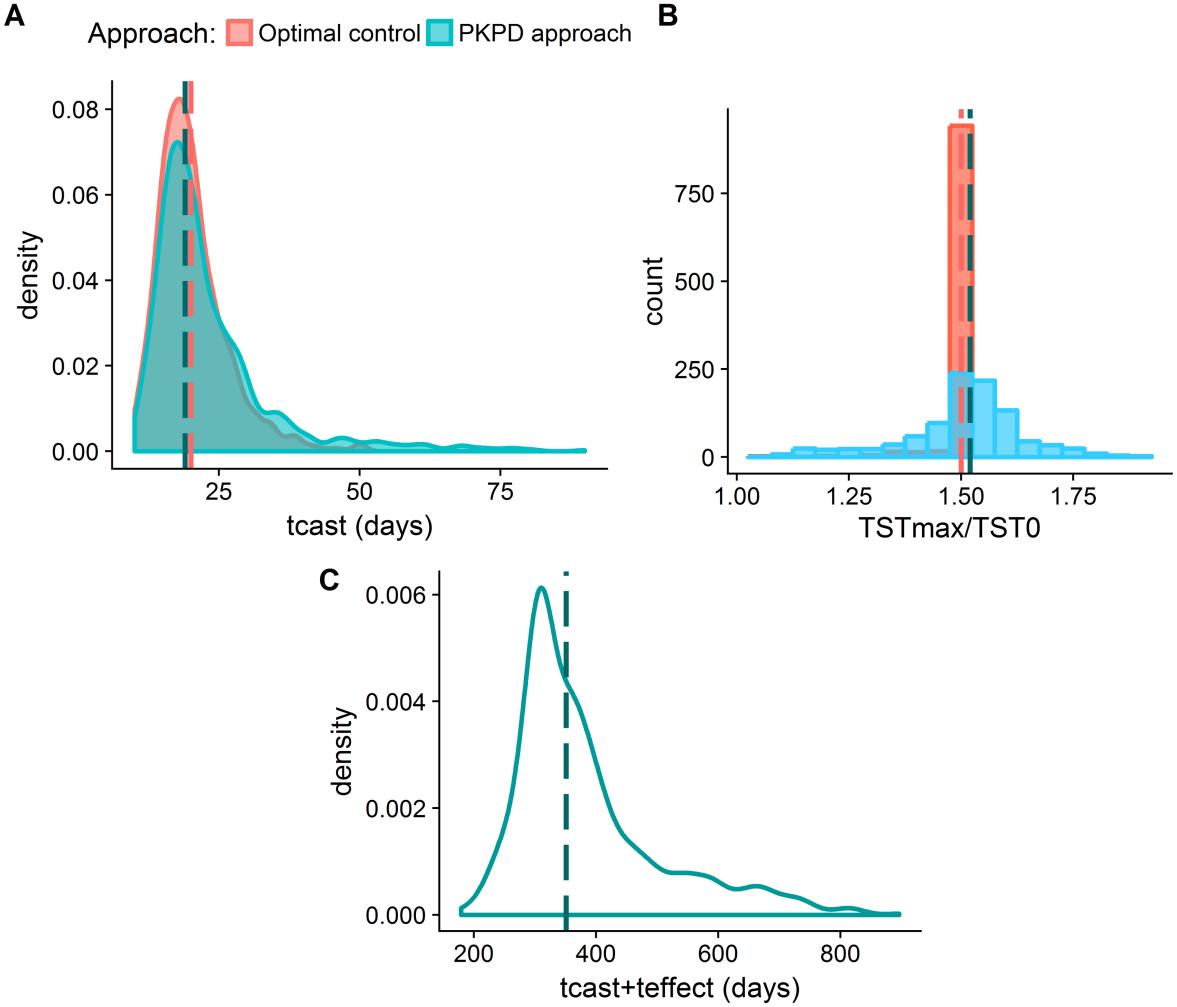
Comparison of the three therapeutic objectives between the optimal control strategy (salmon) and the pharmacokinetic/pharmacodynamic modeling approach (blue) for 1000 individuals. A) Distribution of t_cast_ (time to obtain testosterone levels below castration limit) values. B) Distribution of the values of the testosterone flare-up (maximum testosterone level/baseline testosterone level). C) Distribution of t_cast_ + t_effect_ (castration time after injection of the drug) time values of the modelling approach.

The therapeutic objectives obtained were compared to those from the optimal TST profiles (Fig 7) and the 95% confidence intervals were also calculated from the 1000 samples. Minimal time to achieve castration levels was 19.5 days (11.4-56.7) and the median percentage of drug consumed until that moment was 38.9% (16.55%-66.96%). In Fig 7A the distribution of t_cast_ values for the 1000 individuals can be appreciated. With the modeling approach 63.9% of the patients had a t_cast_ smaller than 21 days, whereas in the optimal TST profiles this value was equal to 70.7%. Regarding the second therapeutic goal, the initial peak in the TST levels had a median of 55% (17%-75%) increase with respect to baseline. This indicated that the second objective was not always achieved, contrary to the case of the TST profiles obtained by the optimal control problem where the flare up was much more controlled (Fig 7B). Nonetheless, the median value was very close to the optimal value of 50%, so we assumed that the modeling approach managed to achieve the second therapeutic goal as well. Finally, the long-term castration had a median value of 351 days (235.9—708), which was higher than expected (Fig 7C), but we again needed to take into account that a small fraction of individuals (7.8%) did not achieve a t_cast_ + t_effect_ above 9 months due to their specific physiological characteristics.

## Discussion

In this paper, we have applied mathematical modeling and control theory to establish optimal drug input (release) profiles to support the development of new release formulation of triptorelin aiming to improve patient coverage. Optimal control (OC) has a long and successful history of applications in engineering [8, 18] and economics [19, 20] but also has become an important issue in biomedical research. Especially in clinical cancer research, a significant amount of effort has been devoted to developing mathematical models to identify the most effective chemotherapeutic administration regimens using OC methods [15, 21] and references therein. One of the earliest studies where chemotherapy treatment planning was defined as an OC problem was in the work from [22]. The authors applied OC to improve the treatment administration of the bone cancer IgG multiple myeloma. Understanding the dynamics of resistance mechanisms against chemotherapy and targeted drugs and emerging of adverse effects represent challenges that have also been addressed through these techniques as shown in the works from [23–26]. For example, in [24] the authors added the pharmacokinetics of the drug in the OC problem in order to provide chemotherapeutic protocols in qualitative terms. The injected drug concentration is used as the control variable and the minimization of the number of tumor cells at the end of the treatment is defined as the cost function of the problem. The results showed that the best strategy corresponds to the maximum rate of drug injection when growth rate is assumed to be constant, but not in other type of models.

Combination of different active compounds, are the rule rather than the exception in oncology and other therapeutic areas, but the potentially high number of different possible combinations (including different dosing schemes), makes drug selection an unaffordable task from an experimental trial and error perspective. Therefore any guide on how to administer these therapies to achieve the best possible responses is of great potential as shown by [27, 28].

In the area of infectious diseases, the works from [29] and [30] showed that using treatment regimens obtained from optimal control could lead to a substantial improvement in HIV patients outcome in comparison to the administration of constant-dose standard regimens. Anesthesia is another medical area where optimal control strategies are used to maintain patient response within the desired therapeutic window for the case of the Bispectral Index (BIS) as an indicator of sedation, or the degree of neuromuscular blockade [31, 32].

Despite the approach shown in the current evaluation is not novel in the drug delivery arena, it has been seldom used beyond optimizing drug exposure. Especially in the context of multi-objective optimization, the current example and others presented below indicate that the optimal control approach should be considered part of the computational modeling arsenal advocated by the FDA promoted critical pathway initiative under the Model Informed Drug Discovery and Development (MID3) paradigm [33].

Here we focused on the non-trivial problem of simultaneously achieving multiple therapeutic goals related to drug onset and offset in the context of clinical trials with a minimum duration of 9 months, which implies high cost and uncertainty regarding the final response outcome. Therefore the possibility of providing to pharmaceutical technology scientists guidance in the form of release/input (absorption) profiles represents a real added value to avoid failed clinical studies. In this context we performed a reverse engineering exercise interpreting the empirical input profiles from a mechanistic biopharmaceutic perspective and showing the practical application of our optimal control analysis, encouraging cooperation between computational and experimental/technology scientists.

In the current exercise the majority of the system accounting for the relationship between dose and response (drug disposition, receptor interaction, and down-regulation mechanisms) was already well characterized with the corresponding typical values and associated variability reported in [3]. In addition the subcutaneous route of administration represents a much simpler biological system compared for example with the oral route. Therefore the optimal control approach used here represents an appropriate choice even recognizing that in more complex situations the advantages offered by similar approaches like non-linear model predictive control [13, 34] could represent a better alternative.

In this context, the main critical aspect of the analysis is the choice of the appropriate structure of the cost function to be minimized and the constraints of the problem. As highlighted in Materials and methods section, we divided the problem into two phases because the minimum time to achieve CT values (t_cast_) in TST levels was not known in advance. For the first phase, we implemented the Mayer form of optimization problems whereas for the second phase a Lagrange term was used (see Table 1). In this work, we focused on the resulting testosterone levels of the prostate cancer patients, but, as shown in the above paragraphs of the Discussion, for other therapeutic areas different objectives could have been established, like the minimization of the tumor cell population at the end of treatment, the maximization of the number of healthy immune system cells or the penalization of excessive application of therapeutic agents [35–37].

The concept of optimization is present at every stage of the drug development process. Optimal design methods, based on the D-optimality criteria which relies on the maximization of the determinant of the Fisher information matrix [38, 39] is becoming also popular to select the appropriate number of subjects in each cohort of the trials, the sampling times and the number of dose levels [40]. However, we must not confuse optimal design methods with optimal control techniques. The aim of the first is to simplify population trials but maintaining the same efficiency as the original studies. There, they do not alter the system equations nor the objective function of the algorithms and the focus is to search for similar results to the original study (identical PKPD parameters, similar concentration vs time profiles…). For example, in the work from [40], they used this method to optimize a population pharmacodynamic experiment of the effect of ivabradine on exercise-induced tachycardia. On the other side, in optimal control, we introduce what are known as control variables into the model equations in order to manipulate the system response towards the desired goal. Therefore, in this type of problems, system equations can be modified and objective functions and constraints defined to search for improved solutions compared to the ones of the original study. Still, both approaches have something in common; they avoid the use of intensive computer simulations when the optimal solutions of a problem are being explored.

### Conclusion

Optimal control theory has been applied to a population pharmacokinetic/pharmacodynamic model to derive the optimal drug release profiles to achieve multiple therapeutic goals. The optimal control analysis is more relevant in physiological systems with complex dynamics where simple simulation tuning parameters exercises are not effective to obtain the optimal profiles. Moreover, the flexibility of the method allows to deal with multiple and tight therapeutic objectives performing real optimization. In this context the question of how to define the objective functions and how to quantify our therapeutic goals becomes crucial. Here, we focused on the resulting testosterone levels of the patients, however, within the oncology area, different therapeutic objectives can be established with the goal of improving drug combinations, help to lessen the side effects of cancer treatments, etc.

Finally, the optimal release characteristics have been described based on standard absorption PK models. Although there are some discrepancies between the resulting TST profiles from the optimal control strategy and the modeling approach (see Results), we note that the important aspect of this work was to find the optimal release characteristics for prostate cancer patients, not to perform an ideal PKPD modeling exercise as there was not real data to fit. We conclude that this objective is achieved and that the information summarized in this article could be very useful for the development of new formulations, since it provides insight into the desired absorption characteristics and could produce a broad benefit for future prostate cancer patients.

## Supporting information

S1 Fig Original pharmacokinetic model of triptorelin. Original pharmacokinetic model of triptorelin from [3]. D_inf_ is the duration of the zero-order absorption process; K_A1_ and K_A2_ are the first order rate constants of the first and second depot compartments respectively; F_1_, F_2_ and F_inf_ represent the fraction of the drug associated with the first and second depot compartments and the zero-order absorption process respectively; t_lag1_/t_lag2_ is the lag time associated to the first/second absorption compartment; C_TRP_ is the serum concentrations of triptorelin; CL, the apparent total clearance; V_c_, V_T1_, and V_T2_, apparent volumes of distribution of the central, shallow, and deep peripheral compartments respectively; and CL_D1_ and CL_D2_, distribution clearances between the central and peripheral compartments.
(TIF)

S1 Text Mathematics behind optimal control methods. Summary of the mathematics involving necessary and sufficient conditions for optimality in dynamic systems.
(PDF)

S1 Data APMonitor MATLAB code and datasets. APMonitor Optimization Suite code for MATLAB and datasets to reproduce the results of this work.
(RAR)
