# Supplementary figures and images for: Optimal dynamic control approach in a multi-objective therapeutic scenario: Application to drug delivery in the treatment of prostate cancer
Source: PLoS Comput Biol. 2018 Apr 19;14(4):e1006087. doi: 10.1371/journal.pcbi.1006087 (PMC5929575; doi:10.1371/journal.pcbi.1006087)

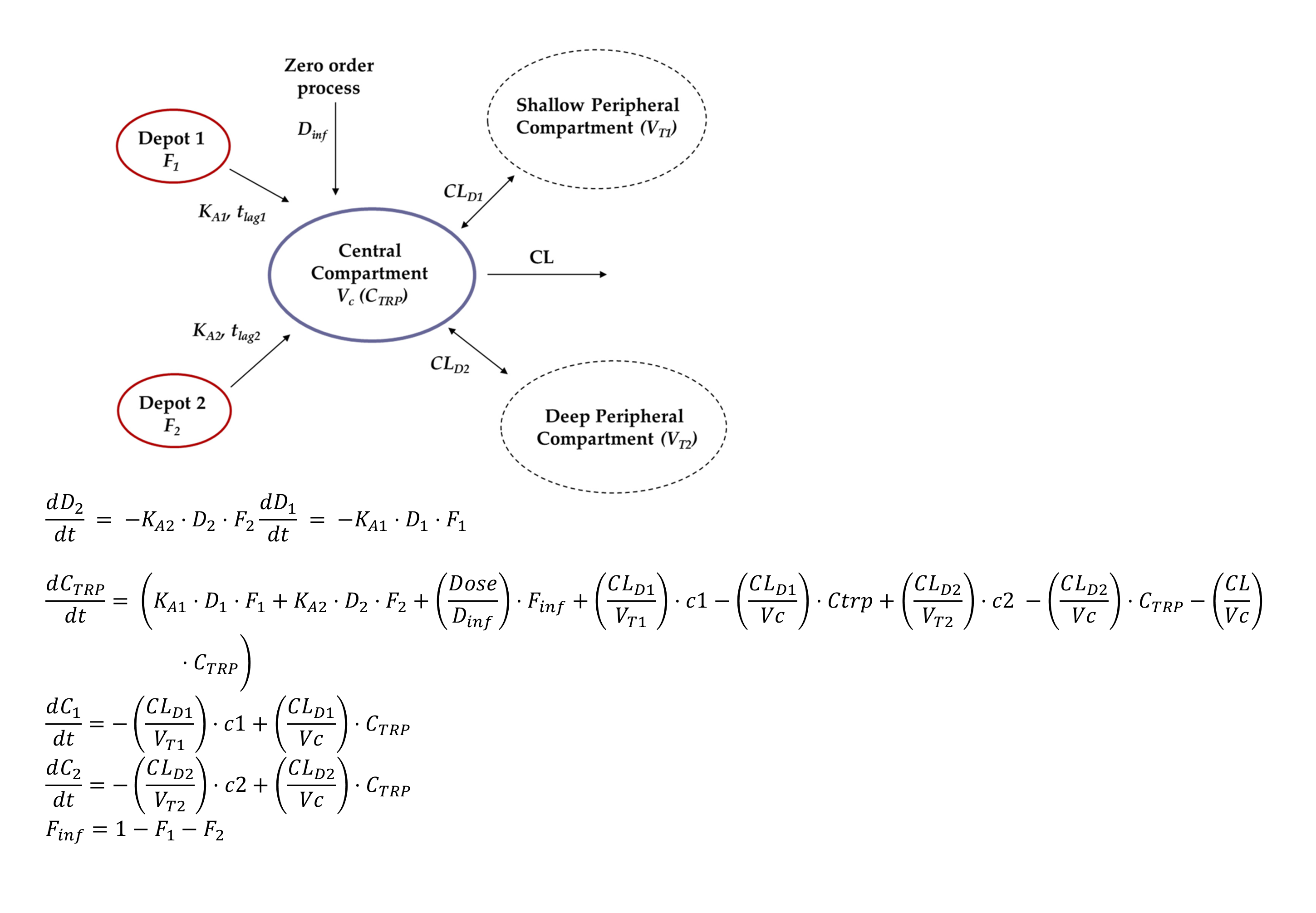

Supplement: S1 Fig — Original pharmacokinetic model of triptorelin from [3]. Dinf is the duration of the zero-order absorption process; KA1 and KA2 are the first order rate constants of the first and second depot compartments respectively; F1, F2 and Finf represent the fraction of the drug associated with the first and second depot compartments and the zero-order absorption process respectively; tlag1/tlag2 is the lag time associated to the first/second absorption compartment; CTRP is the serum concentrations of triptorelin; CL, the apparent total clearance; Vc, VT1, and VT2, apparent volumes of distribution of the central, shallow, and deep peripheral compartments respectively; and CLD1 and CLD2, distribution clearances between the central and peripheral compartments. (TIF) [file pcbi.1006087.s001.tif]
