# Supplementary material for: Optimal dynamic control approach in a multi-objective therapeutic scenario: Application to drug delivery in the treatment of prostate cancer
Source: PLoS Comput Biol. 2018 Apr 19;14(4):e1006087. doi: 10.1371/journal.pcbi.1006087 (PMC5929575; doi:10.1371/journal.pcbi.1006087)
Supplement: S1 Text — Summary of the mathematics involving necessary and sufficient conditions for optimality in dynamic systems. (PDF) [file pcbi.1006087.s002.pdf]

Supplementary material I

Here, we wanted to summarize the mathematics involving the process of finding optimal control histories for dynamic systems. These methods are analogous to the static optimizations, but the computations are necessarily more complex. Additionally, we consider the method of Pontryagin maximum principle to address optimal control problems having path constraints.

1. DYNAMIC SYSTEMS

In dynamic systems, state equations that describe the system and controls are expected to evolve over time and not necessarily remain at a steady state.

Consider the dynamic system described by the ordinary differential equation of the form:

$$\dot{x}(t) = f(x(t), u(t)), \qquad \mathrm{x}(t_0) = x_0 \tag{i}$$

given the control variable $u(t)$ for $t_0 \leq t \leq t_f$ and the following cost function over the time interval $[t_0\ , t_f]$:

$$J = \varphi\left[x\left(t_f\right)\right] + \int_{t_0}^{tf} L[x(t), u(t)]\ dt \tag{ii}$$

The first term of the cost function ii is known as Mayer problem and is a scalar algebraic function of the final state and time and works as a terminal penalty. It is used to assure that one or more state variables satisfy some condition at the end of the trajectory. The second term is known as Lagrange problem and is an integral function of the state and control from t_0 to t_f. When both terms are considered in the cost function, the problem is known as Bolza and its general form is shown in equation ii (Stengel 1986; Betts 2001).

In optimal control problems, the control history $u(t)$ that minimizes J is to be found, subject to specific constraints.

2. DYNAMIC OPTIMIZATION WITH DYNAMIC CONSTRAINTS

As previously described, generally dynamic constraints take the form of ordinary differential equations.

To guarantee that the dynamic constraint is considered in the minimization of a cost function, is has to be adjoined to the integrand term of the equation in such a way that the numerical value of the cost function remains unchanged at its optimum.

Note that in **static minimization**, an equality constraint of the form f(x,u)=0 can be adjoined to the cost function J using Lagrange multipliers (vector λ) to form the augmented cost function J_A:

$$J_A = J + \lambda^T f(x, u) \tag{iii}$$

J_A is identical to J at the minimum because f(x,u) must be zero at that point.

In dynamic optimization however, the equality constraint is derived from the system's differential equation $\dot{x}(t) = f(x(t), u(t))$, which is equivalent to $f(x(t), u(t)) - \dot{x}(t) = 0$. If we convert the constrained optimal control problem into an unconstrained optimal control problem using the Lagrange multipliers, the augmented cost function J_A has the following form:

$$J_A = \varphi[x(t_f)] + \int_{t0}^{tf} \{L[x(t), u(t)] + \lambda^T(t)[f[x(t), u(t)] - \dot{x}(t)]\}\, dt \qquad \text{(iv)}$$

This motivates the construction of the Hamiltonian function defined as

$$H[x(t), u(t), \lambda(t)] = L[x(t), u(t)] + \lambda^T(t) f[x(t), u(t)] \qquad \text{(v)}$$

Thus,

$$J = \varphi[x(t_f)] + \int_{t_0}^{tf} \{H[x(t), u(t), \lambda(t)] - \lambda^T(t)\, \dot{x}(t)\}\, dt \qquad \text{(vi)}$$

Now J_A depends on $\dot{x}(t)$. Using integration by parts,

$$\int_{t_0}^{t_f} \lambda^T(t)\, \dot{x}(t) = \lambda^T(t_f) x(t_f) - \lambda^T(t_0) x(t_0) - \int_{t_0}^{t_f} \dot{\lambda}^T(t) x(t) \qquad \text{(vii)}$$

the cost function becomes

$$J_A = \varphi[x(t_f)] + [\lambda^T(t_0) x(t_0) - \lambda^T(t_f) x(t_f)] + \int_{t_0}^{tf} \{H[x(t), u(t), \lambda(t)] + \dot{\lambda}^T(t)\, x(t)\}\, dt \qquad \text{(viii)}$$

From this point, the augmented cost function will be written simply as J.

3. CONDITIONS FOR OPTIMALITY

One of the main issues in optimal control is to assure whether an optimal control exists for a given problem. In the next section, we describe a set of conditions which any optimal control must satisfy.

3.1 Necessary conditions for optimality

Necessary conditions indicate that the cost function should be insensitive to small control variations (Δu) in the optimum point. This is mathematically expressed as $\Delta J^*[u*, \Delta u] = 0$ (the first variation of J due to small variation in u must be equal 0). Setting the terms that multiply these variations to be zero yields to the three necessary conditions for optimality, known as the Euler-Lagrange equations (described in detail in (Stengel 1986)):

- $\left[\frac{\partial \varphi}{\partial x} - \lambda^T\right]_{t=t_f} = 0$ (ix)
- $\left[\frac{\partial H}{\partial x} + \dot{\lambda}^T\right] = 0 \quad in\ (t_0, t_f)$ (x)
- $\frac{\partial H}{\partial u} = 0\ \ in\ (t_0, t_f)$ (ix)

Note that, in this section, we consider optimal control problems having no restriction on the control variables.

3.2 Sufficient conditions for optimality

In many optimal control problems, cost stationarity may imply optimality because there is sufficient knowledge of the system and flexibility in the choice of the cost function. Even so, in (Stengel 1986) three sufficient conditions are described (convexity condition, normality condition and uniqueness or Jacobi condition), which along with the necessary conditions, would assure optimality. The most important one (and the only condition that is discussed for this work) is the convexity condition, also known as Legendre-Clebsch condition:

$$\frac{\partial^2 H(x^*, u^*, \lambda^*)}{\partial u^2} > 0 \quad in\ (t_0, t_f) \qquad \text{(xii)}$$

Where x*, u* and λ* refer to the optimal state, control and Lagrange variables found as solution to the problem respectively. This property implies that every stationary point in u is a strict local minimizer of the Hamiltonian H in the control. Using quadratic formulations in the cost function considerably simplifies the analysis of this condition because the Hamiltonian becomes convex in the control u and thus has a unique minimizer. While this does not guarantee that the controls found by the analysis are necessarily optimal, it introduces important mathematical advantages (Schättler and Ledzewicz 2015).

3.3 Pontryagin's Principle

In this section, we present more general necessary conditions for optimality for those problems having bounded control magnitudes. Such conditions were generalized by Pontryagin and co-workers in their Maximum principle (sometimes also referred as Pontryagin's Minimum Principle) (Pontryagin et al. 1962).

Because of the restriction on u, the necessary condition of section 3.1 changes to $\Delta J^*[u^*, \Delta u] \geq 0$. This implies that

$$H[x^*(t), u^*(t), \lambda^*(t)] \leq H[x^*(t), u^*(t) + \Delta u, \lambda^*(t)] \qquad \text{(xiii)}$$

Equation xiii is the necessary conditions stated by Pontryagin in his theorem, which along with equations ix and x, state the necessary conditions for optimality when control variables are subject to constraints on their magnitudes. The maximum principle can in some cases provide a solution to problems where the Euler-Lagrange equations fail. One example is when linear term are used in the cost function and the equation $\frac{dH}{du}$ becomes identically zero.

Both Euler-Lagrange equations and the maximum principle are difficult to apply to large non-linear systems with state or control variable constraints and this may lead to computationally intractable problems. In these cases, where an analytical solution is not possible to be found, computational algorithms are inevitable in solving optimal control problems (Hedengren et al. 2014).

References:

Betts, John T. 2001. *Practical Methods for Optimal Control Using Nonlinear Programming*. Society for Industrial and Applied Mathematics.

Hedengren, John D., Reza Asgharzadeh Shishavan, Kody M. Powell, and Thomas F. Edgar. 2014. “Nonlinear Modeling, Estimation and Predictive Control in APMonitor.” *Computers & Chemical Engineering* 70 (November): 133–48.

Pontryagin, L. S., V. G. Boltyanskii, R. V. Gamkrelidze, and E. Mishchenko. 1962. “The Mathematical Theory of Optimal Processes (International Series of Monographs in Pure and Applied Mathematics).” *Interscience, New York*.

Schättler, Heinz, and Urszula Ledzewicz. 2015. *Optimal Control for Mathematical Models of Cancer Therapies: An Application of Geometric Methods*. Interdisciplinary Applied Mathematics 42. Springer New York.

Stengel, Robert F. 1986. *Optimal Control and Estimation*. Courier Corporation.
